# Shaping the Future of Pharmacology Education: Emerging Directions and International Initiatives From the Seventh International Union of Basic and Clinical Pharmacology Education Satellite Meeting

**DOI:** 10.1002/prp2.70338

**Published:** 2026-10-25

**Authors:** Clare Guilding, Anna‐Marie Babey, Kellie Charles, Margaret Cunningham, Betty Exintaris, Lynette B. Fernandes, Tina Hinton, Nilushi Karunaratne, Jennifer Koenig, Graham Mackay, Carolina Restini, John L. Szarek, Paul J. White, Kelly Karpa

**Affiliations:** ^1^ School of Medicine, Faculty of Medical Sciences Newcastle University Newcastle upon Tyne UK; ^2^ School of Science & Technology University of New England Armidale New South Wales Australia; ^3^ Sydney Pharmacy School The University of Sydney Sydney New South Wales Australia; ^4^ Strathclyde Institute of Pharmacy and Biomedical Sciences (SIPBS) University of Strathclyde Glasgow UK; ^5^ Faculty of Pharmacy and Pharmaceutical Sciences Monash University Parkville Victoria Australia; ^6^ School of Biomedical Sciences The University of Western Australia Perth Western Australia Australia; ^7^ Faculty of Medicine and Health, Sydney Pharmacy School The University of Sydney Sydney New South Wales Australia; ^8^ School of Medicine University of Nottingham Nottingham UK; ^9^ Department of Biochemistry and Pharmacology, School of Biomedical Sciences The University of Melbourne Melbourne Victoria Australia; ^10^ College of Osteopathic Medicine, Michigan State University East Lansing Michigan USA; ^11^ Department of Medical Education Geisinger College of Health Sciences School of Medicine Scranton Pennsylvania USA; ^12^ Quillen College of Medicine, East Tennessee State University Johnson City Tennessee USA

**Keywords:** authentic assessment, competency‐based education, curriculum integration, generative artificial intelligence, pharmacology education, professional identity

## Abstract

The 7th International Union of Basic and Clinical Pharmacology Education Section (IUPHAR‐Ed) Satellite Meeting, held in conjunction with the 20th World Congress of Basic and Clinical Pharmacology in Melbourne, Australia, brought together 99 educators from 18 countries to share innovations and scholarship in pharmacology education. Across keynote presentations, workshops, short communications, and posters, four recurring directions emerged: a shift toward competency‐based curricula and authentic assessment; strategies for sustaining pharmacology within integrated curricula; thoughtful incorporation of generative artificial intelligence into teaching and assessment; and broader expectations for pharmacologists to engage with the ethical, cultural, environmental, and societal dimensions of medicines. Together, these themes reflect an evolution in pharmacology education from emphasizing knowledge acquisition toward preparing adaptable professionals who can apply pharmacological science through critical thinking, collaboration, and professional judgment. The meeting also highlighted the value of international collaboration and shared educational resources in advancing innovation across diverse institutional and educational contexts. These observations provide insight into priorities that may shape the future of pharmacology education worldwide.

**Abbreviations**
AI: artificial intelligence
IUHAR: International Union of Basic and Clinical Pharmacology
IUHAR‐Ed: International Union of Basic and Clinical Pharmacology Education Section
PEP: Pharmacology Education Project
TGA: Therapeutic Goods Administration
WCP: World Congress of Basic and Clinical Pharmacology

## 1 Introduction

The International Union of Basic and Clinical Pharmacology Education Section (IUPHAR‐Ed) convened its 7th Satellite Meeting alongside the 20th World Congress of Basic and Clinical Pharmacology (WCP). Held every 4 years, the IUPHAR‐Ed Satellite Meeting brings together pharmacology educators from around the world to share innovative practices, scholarship, and research in undergraduate and postgraduate education across the biosciences and health professions. Hosted on July 11–12, 2026, by Monash University Faculty of Pharmacy and Pharmaceutical Sciences in Melbourne, Australia, the event was attended by 99 individuals from 18 countries and 52 institutions (Figure 1).

**FIGURE 1:**
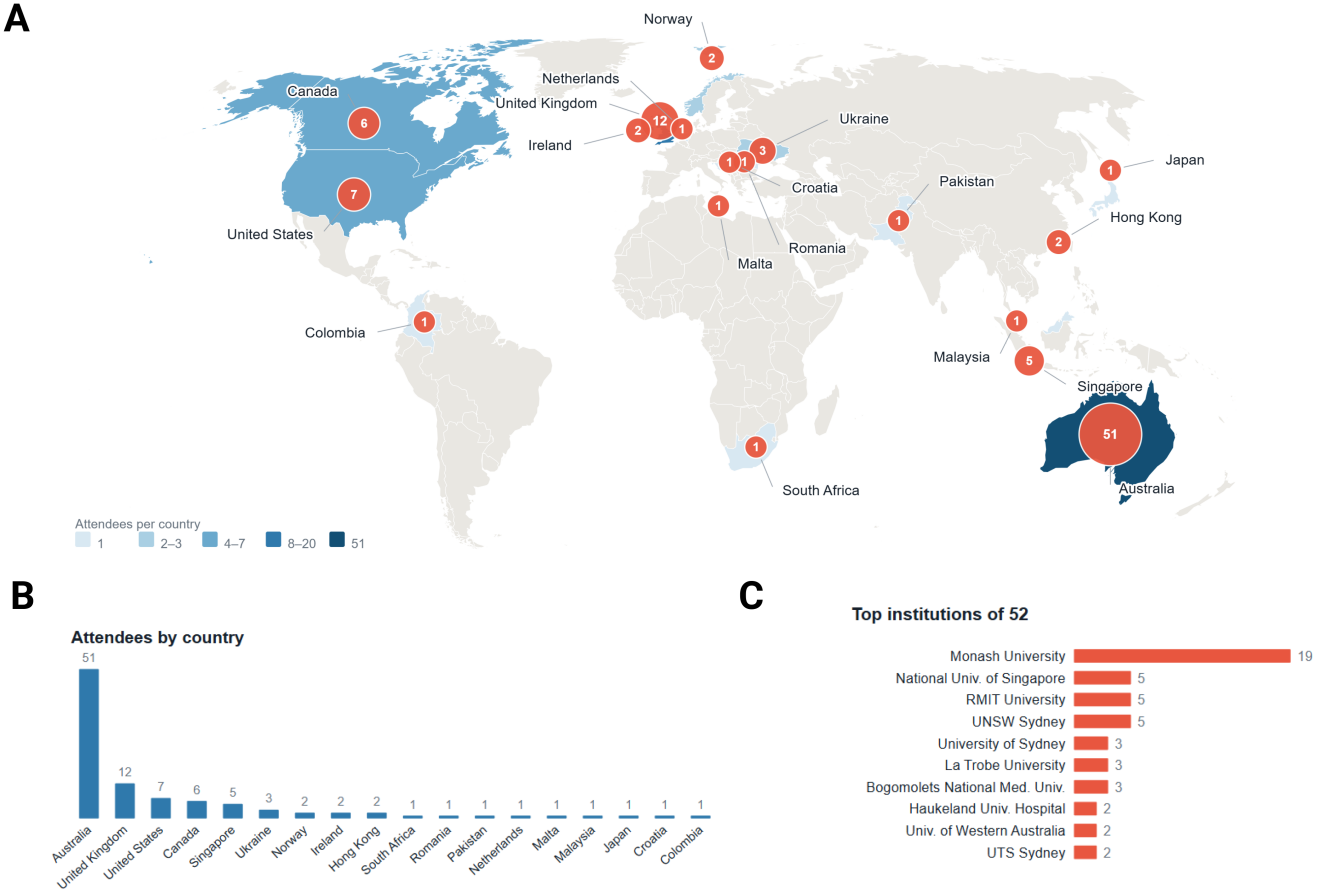
Geographic distribution of attendees at the 7th IUPHAR‐Ed Satellite Meeting, based on attendee affiliation data exported from the conference registration system. Country and institutional data (Panels A and B) were standardized and aggregated prior to visualization using Python (GeoPandas and Matplotlib) with a Natural Earth basemap. Panel C depicts institutions with the most attendees.

Across keynote lectures, rapid‐fire presentations, short talks, workshops, and posters, contributors explored a wide range of educational contexts and challenges [1]. Several recurring themes nevertheless emerged, providing insight into the ongoing evolution of pharmacology education. The presentations reflected a movement toward competency‐based curricula and authentic assessment, integrated curricular models, responsible use of artificial intelligence (AI), and expanded conceptions of a pharmacologist's role in society. Together, these shifts suggest an evolving international vision of pharmacology education that emphasizes not only what learners know but how effectively they can apply pharmacological knowledge in professional, scientific, and societal contexts [2].

## 2 Methods

Prior to the satellite meeting, authors developed a structured note‐taking strategy to enhance coverage across the program. One author (K.K.) took notes through the entire meeting to provide continuity across sessions. Additional authors were assigned in advance as primary and secondary note‐takers for specific program sections, with secondary note‐takers reviewing and supplementing the primary notes. Following the meeting, notes were collated and reviewed and were subsequently provided to ChatGPT (GPT‐5.6; OpenAI, San Francisco, CA), which was used as an organizational aid to identify recurring ideas and propose initial thematic groups. AI‐generated groupings were not accepted as final themes. Authors reviewed the suggested groupings against the meeting notes, merged overlapping concepts, reframed themes where appropriate, and determined the final thematic structure. These themes presented here represent an interpretive synthesis of the meeting rather than a formal qualitative analysis or consensus statement of IUPHAR‐Ed.

## 3 Results

Four recurring themes were identified from presentations and discussions and are described below.

### 3.1 Emerging Direction 1: Pharmacology Education Is Shifting Toward Competency‐Based Learning and Authentic Assessment

The strongest theme emerging from the meeting was a shift from curricula centered primarily on knowledge acquisition toward curricula preparing graduates with the knowledge, skills, and professional attributes required for increasingly diverse careers in academia, industry, government, and healthcare [2]. This perspective was exemplified by David Lewis, recipient of the Elizabeth Davis Teaching Excellence Award, whose keynote address argued that traditional didactic approaches alone are insufficient for contemporary pharmacology graduates. He presented educational approaches that combine experiential, enquiry‐based, and collaborative learning with authentic assessment to develop the knowledge, skills, and professional competencies required for an increasingly diverse range of careers [3] (Table 1). This perspective was reinforced throughout the meeting by presentations describing competency frameworks, outcome‐based prescribing curricula, workplace‐aligned assessment strategies, and international efforts to identify enduring concepts that underpin pharmacology education. These presentations illustrated a growing international emphasis on preparing graduates to apply pharmacological knowledge in complex professional environments.

**TABLE 1:** Examples of authentic assessment activities.

Examples	Presenting university	Brief description
Acting as drug regulators	University of Melbourne	Students assume the role of Australia's Therapeutic Goods Administration (TGA), reviewing preclinical data, evaluating safety and efficacy, and making approval decisions about real and fictional drugs
Preparing Institutional Animal Care and Use Committee (IACUC)‐ type submissions/Animal Ethics applications	University of Leeds	Students critique flawed animal ethics applications and prepare their own regulatory submissions addressing scientific, ethical, legal, and welfare issues
Debating Animal Ethics	University of Leeds	Students role‐play animal activists, animal welfare advocates, researchers from affluent and low‐income countries while debating use of animal models in drug discovery
Workplace projects/work‐based outputs	University of Leeds	Students produce grant proposals, policy documents, academic papers, business reports, and e‐portfolios designed to showcase workplace competencies
DrugBank biocuration	University of Alberta	Students partner with DrugBank to curate pharmacokinetic/pharmacogenomic information from primary literature, evaluate AI‐generated information, and synthesize evidence into authentic drug dossiers
Regulatory Briefing Notes	University of Leeds	Students complete an assignment based on ICH S7A (*Safety Pharmacology Studies for Human Pharmaceuticals*), an international regulatory guideline outlining nonclinical testing used to identify undesirable pharmacodynamic effects of new drug candidates on vital physiological functions before human studies
Objective Structured Practical Examinations (OSPEs)	Monash University	Students complete stations assessing laboratory skills, interpretation of data, verbal defense of decisions, and explanation of concepts
Inquiry Projects	Swansea University	Students generate original experimental data using *Lumbriculus variegatus* , participate in blinded multicenter studies, and analyze their own findings

This shift in educational goals has important implications for assessment. If curricula prepare graduates to apply pharmacological knowledge in professional contexts, assessments must also evaluate how learners reason, make decisions, and perform authentic professional tasks. Educators described assessments in which students assume professional roles, make evidence‐informed decisions, defend their reasoning, and produce deliverables like those expected in the workplace (Table 1). Additionally, presentations highlighted that assessment design should be inclusive, flexible, and equitable, reflecting the unique strengths, needs, and backgrounds of diverse learners. This ensures opportunities for all students to demonstrate their true knowledge, skills, and potential while respecting individual differences. Together, these approaches shift assessment from measuring *what* students know to *how* they demonstrate pharmacological knowledge in practice.

These changes in curricular goals and assessment also reshape the learner's role. Demonstrating competence in professional contexts requires students to move beyond passive information acquisition to active participation in knowledge construction, inquiry, and professional development. Project‐based curricula encourage students to assume roles within collaborative teams, develop project outputs with real‐world relevance, participate in peer review and assessment, and contribute to solving complex scientific problems. For example, students at Swansea University participate in multicenter collaborative research projects in which they generate original experimental data, contribute to shared datasets, and engage in scientific inquiry under the direction of Outstanding Early Career Education Award recipient Aiden Seeley [4]. Other presentations highlighted learner involvement in developing educational resources, critically evaluating AI‐generated content, and selecting assessment pathways aligned with individual interests and career goals. These learner‐centered approaches align with broader trends in health professions education that emphasize agency, self‐directed learning, collaboration, and professional identity formation [5].

Taken together, these developments represent interconnected dimensions of the same educational shift: competency‐based curricula define what graduates should be able to do, authentic inclusive assessments determine whether they can do it, and learner‐centered pedagogies provide the experiences through which those capabilities are developed. This evolution positions learners as active participants in their own professional development and supports the preparation of graduates who are reflective, adaptable, and practice ready.

### 3.2 Emerging Direction 2: Pharmacology Education Is Moving From Integration to Implementation

A second theme occurring across presentations and workshop discussions was that many institutions have moved beyond advocating for integrated curricula and are now focused on implementation. Rather than debating whether pharmacology should be integrated into systems‐based health professions curricula, presenters focused on the practical challenges of ensuring that the discipline remains coherent, visible, and educationally meaningful within increasingly complex curricular structures. Educators identified common challenges, including fragmentation of content across organ systems, inconsistent sequencing of foundational concepts, redundancy without purposeful reinforcement, variable faculty expertise, and assessments that inadequately evaluate pharmacological reasoning [6]. Discussions emphasized the importance of deliberate curricular design to maintain coherence and progression in pharmacology learning. Proposed solutions included intentional sequencing, faculty development, clearly articulated learning outcomes, integrated assessment, and ongoing curriculum mapping. International initiatives such as the IUPHAR Core Concepts Project support these efforts by providing a common framework for curriculum mapping, longitudinal reinforcement, and maintaining the visibility of pharmacology within health professions education [7]. Collectively, these discussions suggest that the central challenge is no longer whether pharmacology should be integrated, but how integration can be achieved while preserving disciplinary coherence, longitudinal development, and the identity of pharmacology within the curriculum.

### 3.3 Emerging Direction 3: Generative AI Is Catalyzing the Redesign of Pharmacology Education

Generative artificial intelligence (AI) emerged as a recurring theme, not as a replacement for educators, but as a catalyst for rethinking how pharmacology is taught, learned, and assessed. Rather than focusing primarily on academic integrity, discussions emphasized that the widespread availability of AI‐generated responses requires assessment approaches that reveal and evaluate students' reasoning, conceptual understanding, and professional judgment.

Several presentations illustrated how AI can be integrated thoughtfully into teaching and assessment (Table 2). Faculty from Macquarie University described a reflective AI‐enhanced assessment in which students compared their own responses with AI‐generated answers, identified misconceptions, consulted the literature, and refined their understanding. Colleagues from the National University of Singapore presented an educator‐vetted workflow that uses AI to generate flashcards and formative assessment questions aligned with learning objectives and Bloom's taxonomy while emphasizing the importance of faculty oversight to ensure accuracy and pedagogical alignment. Student feedback suggested that these resources improved understanding and confidence, although presenters noted that flashcards alone are unlikely to promote higher‐order learning without opportunities for application and reasoning.

**TABLE 2:** Examples of Educational and Institutional Approaches to Generative Artificial Intelligence (AI).

Institution	AI‐related innovation	Educational purpose	Broader implication
Macquarie University	Reflective AI‐enhanced assessment	Students critique AI‐generated responses, identify misconceptions, and revise their understanding	AI can promote metacognition when learners critically evaluate rather than simply accept AI output
National University of Singapore	Faculty‐vetted AI‐generated flashcards and formative question banks	Support active recall, spaced repetition, and efficient creation of learning resources	AI improves resource development efficiency but requires faculty oversight
University of Sydney	Need for AI literacy among faculty	Identify institutional supports needed for educators to adopt AI responsibly	Successful AI integration will depend on faculty development and institutional infrastructure, not technology alone

Beyond teaching and assessment, AI was also discussed as a faculty development and institutional challenge. Workshop discussions highlighted the need to build educator AI literacy, provide institutional support, and establish governance strategies for responsible curriculum integration. Faculty leading the Pharmacology Education Project (PEP), IUPHAR's open‐access educational resource, described future directions that may include AI‐assisted content development supported by expert editorial oversight to maintain scientific accuracy and educational quality [8].

Collectively, these presentations suggest that AI is being positioned as a tool to support learning, reasoning, and professional development rather than simply accelerating information retrieval [9]. The greatest educational impact of AI may lie in prompting educators to redesign learning and assessment around critical thinking, evidence‐based decision making, and authentic professional practice [2].

### 3.4 Emerging Direction 4: Professional Responsibilities of Pharmacologists Are Expanding

A fourth theme reflected an expanding conception of the pharmacologist's role. While scientific expertise remains foundational, presenters emphasized that contemporary pharmacologists must also consider the ethical, cultural, environmental, and societal contexts in which medicines are discovered, developed, prescribed, and used. Across keynote presentations, workshops, and short communications, topics such as communication, teamwork, equity, cultural responsiveness, and sustainability were presented not as peripheral additions to pharmacology education, but as integral components of professional practice.

One dimension of this broader perspective involves recognizing how biological variation and social context influence pharmacological decision‐making. For example, a workshop on ethnicity in pharmacology education challenged participants to critically examine how race and ethnicity are represented in teaching and clinical decision‐making, highlighting the limitations of using these constructs as proxies for genetic variation [10].

Attention also extended to the environmental impacts of medicines [11]. During a session on environmental stewardship, presenters introduced principles of green and sustainable pharmacology using the Understanding by Design (UbD) framework and explored how medicines affect the environment throughout their lifecycle, from manufacture to disposal [12]. Presenters also described PlanED Prescribing, a multi‐institutional European initiative that integrates planetary health into prescribing curricula through authentic clinical cases, encouraging students to consider environmental impact alongside efficacy, safety, and cost [13]. Discussions further emphasized the importance of decolonization, diversity, equity, and inclusion, encouraging educators to examine whose perspectives are represented in curricula and address barriers to participation in global pharmacology education.

Collectively, these presentations point toward a broader model of pharmacology education that integrates environmental stewardship, sustainable prescribing, equity, cultural relevance, and critical engagement with emerging technologies. Achieving this vision will require interdisciplinary collaboration, accessible educational resources, and sustained attention to the structural barriers that influence implementation.

## 4 Discussion

The presentations and discussions at the 7th IUPHAR‐Ed Satellite Meeting suggested a broader evolution in the aims of pharmacology education. While pharmacological knowledge remains foundational, educational success is increasingly defined by learners' abilities to reason, collaborate, exercise professional judgment, and apply knowledge effectively in practice.

This evolution has important implications for curriculum design, teaching, and assessment. Competency‐based curricula, inclusive assessment, learner agency, curricular integration, and broader professional responsibility are not isolated trends but interconnected components of a practice‐oriented approach to education. Preparing learners for contemporary professional environments requires alignment among learning outcomes, educational experiences, and assessment.

Generative AI reinforces this shift by exposing the limitations of approaches that primarily reward information retrieval or reproduction. As access to AI‐generated information becomes routine, educators will increasingly need to emphasize critical evaluation, recognition of uncertainty, justification of decisions, and appropriate application of pharmacological principles. AI may therefore accelerate existing movements toward higher‐order reasoning, authentic assessment, and professional competence.

For pharmacology educators, adapting these changes may require attention to their own professional development. Educators may need to develop greater fluency with AI and emerging educational technologies, strengthen skills in authentic and competency‐based assessments, and engage with topics like cultural responsiveness and environmental stewardship that may extend beyond their traditional disciplinary expertise. Networks of educators, professional societies, and international collaborations can facilitate adaptation through exchange of resources, approaches, and expertise that may be difficult for individual educators or institutions to develop independently. Importantly, implementing these changes need not require wholesale curricular redesign; many approaches highlighted at the meeting can be incorporated by reframing existing learning activities and assessments.

The themes described here reflect presentations and discussions at a single international meeting and should not be interpreted as a comprehensive survey of pharmacology education worldwide. Rather, they represent recurring priorities identified by educators from diverse institutional and national contexts that may warrant continued exploration and evaluation.

Overall, the meeting reflected a shift from viewing pharmacology education primarily as knowledge acquisition toward developing professionals who can apply, evaluate, and communicate pharmacological knowledge responsibly within increasingly complex scientific, clinical, and societal contexts.

## Author Contributions

**Clare Guilding:** writing – review and editing, writing – original draft. **Anna‐Marie Babey:** writing – review and editing. **Kellie Charles:** writing – review and editing, writing – original draft. **Margaret Cunningham:** writing – review and editing, visualization. **Betty Exintaris:** writing – review and editing. **Lynette B. Fernandes:** writing – review and editing, writing – original draft. **Tina Hinton:** writing – review and editing, writing – original draft. **Nilushi Karunaratne:** writing – review and editing. **Jennifer Koenig:** writing – review and editing. **Graham Mackay:** writing – review and editing, writing – original draft. **Carolina Restini:** writing – review and editing, writing – original draft. **John L. Szarek:** writing – review and editing, writing – original draft. **Paul J. White:** writing – review and editing. **Kelly Karpa:** conceptualization, writing – original draft, writing – review and editing.

## Funding

The authors have nothing to report.

## Conflicts of Interest

The authors declare no conflicts of interest.

## Data Availability

The data that support the findings of this study are available from the corresponding author upon reasonable request.

## References

[1] P. White , C. Guilding , M. Cunningham , et al., 7th Annual WCP IUPHAR Education Section Satellite Conference (Frontiers Media SA, 2026), 10.3389/978-2-8325-8037-0.

[2] C. Guilding , R. Kelly‐Laubscher , M. Cunningham , et al., “Reimagining Pharmacology Education,” Pharmacological Reviews 78, no. 3 (2026): 100126, 10.1016/j.pharmr.2026.100126. PMC13197921 42019093

[3] D. I. Lewis , “Final Year or Honours Research Projects: Time for a Total Re‐Think?” (2020) *Physiology News*, 119. The Physiological Society, https://www.physoc.org/magazine‐articles/final‐year‐or‐honours‐research‐projects/.

[4] J. J. Carriere , N. A. Davies , M. R. Cunningham , M. J. Wallace , and A. Seeley , “Co‐Created In Vivo Pharmacology Practical Classes Using the Novel Organism *Lumbriculus variegatus* ,” Pharmacology Research & Perspectives 11, no. 6 (2023): e01158, 10.1002/prp2.1158. PMC10704400 38063050

[5] M. H. Murad , F. Coto‐Yglesias , P. Varkey , L. J. Prokop , and A. L. Murad , “The Effectiveness of Self‐Directed Learning in Health Professions Education: A Systematic Review,” Medical Education 44, no. 11 (2010): 1057–1068, 10.1111/j.1365-2923.2010.03750.x. 20946476

[6] P. S. Fasinu and T. W. Wilborn , “Pharmacology Education in the Medical Curriculum: Challenges and Opportunities for Improvement,” Pharmacology Research & Perspectives 12, no. 1 (2024): e1178, 10.1002/prp2.1178. PMC10869893 38361337

[7] P. J. White , C. Guilding , T. Angelo , et al., “Identifying the Core Concepts of Pharmacology Education: A Global Initiative,” British Journal of Pharmacology 180, no. 9 (2023): 1197–1209, 10.1111/bph.16000. 36470846

[8] J. Szarek , C. Guilding , and S. Maxwell , “Transforming Pharmacology Education: Insights From the Pharmacology Education Project in the Era of Digital Learning,” European Journal of Pharmacology 989 (2025): 177258, 10.1016/j.ejphar.2025.177258. 39793880

[9] W. Cordier and P. Ijeoma , “Mapping Current Use of Artificial Intelligence in Pharmacology Education via a Scoping Review,” Pharmacology Research & Perspectives (2026), 10.1002/prp2.70290. PMC13322406 42384579

[10] A. H. Goodman , “Why Genes Don't Count (For Racial Differences in Health),” American Journal of Public Health 90, no. 11 (2000): 1699–1702, 10.2105/ajph.90.11.1699. PMC1446406 11076233

[11] G. S. Dawe and R. Rajkumar , “Teaching the True Cost of Medicines. White Paper for a Green and Sustainable Pharmacology Curriculum,” Frontiers in Pharmacology (2026), 10.3389/fphar.2026.1912700/abstract. PMC13662423 42876982

[12] A. D. Newell , C. A. Foldes , A. J. Haddock , N. Ismail , and N. P. Moreno , “Twelve Tips for Using the Understanding by Design Curriculum Planning Framework,” Medical Teacher 46, no. 1 (2024): 34–39, 10.1080/0142159X.2023.2224498. PMC10728343 37334694

[13] J. D. Piët , A. Booth , E. M. Donker , et al., “Environmentally Sustainable Prescribing: Recommendations for EU Pharmaceutical Legislation,” Lancet Planetary Health 8, no. 10 (2024): e715–e716, 10.1016/S2542-5196(24)00230-4. 39303730

